# Supplementary material for: Normalization of the microbiota in patients after treatment for colonic lesions
Source: Microbiome. 2017 Nov 16;5:150. doi: 10.1186/s40168-017-0366-3 (PMC5689185; doi:10.1186/s40168-017-0366-3)
Supplement: Supplementary file 1 — Normalization of the microbiota in patients after treatment for colonic lesions (PDF 31 kb) [file 40168_2017_366_MOESM1_ESM.pdf]

Normalization of the microbiota in patients after treatment for colonic lesions

Marc A Sze, Nielson T Baxter, Mack T Ruffin IV, Mary AM Rogers, and Patrick D Schloss

Supplemental

Table S1: P-values of Differences between Initial and Follow Up Samples for Richness, Shannon Diversity, and Evenness.

Comparison	Alpha Metric	P-value	BH Adjusted P-value
Adenoma	richness	0.2615199	0.2942099
Adenoma	shannon	0.2221074	0.2942099
Adenoma	evenness	0.2347622	0.2942099
Advanced Adenoma	richness	0.1231880	0.2217384
Advanced Adenoma	shannon	0.0204124	0.0774651
Advanced Adenoma	evenness	0.0258217	0.0774651
Carcinoma	richness	0.5822250	0.5822250
Carcinoma	shannon	0.0890290	0.2003153
Carcinoma	evenness	0.0072909	0.0656181

Table S2: Summary of Common OTUs for All Diagnosis Groups

OTU	Lowest Taxonomic ID	Adenoma Median MDA	Advanced Adenoma Median MDA	Carcinoma Median MDA	NA
Otu000015	Lachnospiraceae	OTU15	1.24	0.40	0.54
Otu000012	Clostridiales	OTU12	1.14	1.20	0.35
Otu000605	Blautia	OTU605	0.82	0.52	0.39
Otu000006	Bacteroides	OTU6	0.78	0.57	0.62
Otu000061	Ruminococcaceae	OTU61	0.74	0.29	0.54
Otu000005	Bacteroides	OTU5	0.72	0.47	0.56
Otu000044	Clostridium XlVa	OTU44	0.66	0.80	0.32
Otu000058	Lachnospiraceae	OTU58	0.65	0.44	0.42
Otu000013	Blautia	OTU13	0.63	0.49	0.43
Otu000004	Anaerostipes	OTU4	0.63	0.32	0.62
Otu000009	Blautia	OTU9	0.62	0.30	0.34
Otu000037	Streptococcus	OTU37	0.60	0.53	1.02
Otu000014	Clostridiales	OTU14	0.60	0.38	0.35
Otu000002	Anaerostipes	OTU2	0.54	0.31	0.46
Otu000010	Roseburia	OTU10	0.51	0.27	0.50
Otu000028	Ruminococcus	OTU28	0.49	0.42	0.35
Otu000290	Clostridium IV	OTU290	0.49	0.30	0.38
Otu000001	Bacteroides	OTU1	0.47	0.37	0.66
Otu001307	Coprobacillus	OTU1307	0.46	0.38	0.89
Otu000041	Clostridium XI	OTU41	0.46	0.34	0.43
Otu000003	Blautia	OTU3	0.46	0.31	0.39
Otu000020	Faecalibacterium	OTU20	0.46	0.28	0.33
Otu000059	Bacteroides	OTU59	0.45	0.42	0.40
Otu000016	Dorea	OTU16	0.44	0.36	0.37
Otu000085	Dorea	OTU85	0.41	0.27	0.59
Otu000026	Collinsella	OTU26	0.39	0.33	0.76
Otu000036	Lachnospiraceae	OTU36	0.39	0.27	0.35
Otu000030	Phascolarctobacterium	OTU30	0.39	0.25	0.31

Table S3: Summary of Comparisons Based on Additive Treatment Received (Chemotherapy or Chemotherapy and Radiation)

Variable	Additive Treatment Mean	Removal Mean	Additive Treatment SD	Removal SD	P-value	BH Corrected
Richness	-14.22	-32.99	93.58	111.2	6.6e-01	9.2e-01
Shannon	0.17	0.26	0.55	0.7	9.2e-01	9.2e-01
Evenness	0.03	0.06	0.08	0.1	8.6e-01	9.2e-01
Thetayc	0.80	0.72	0.11	0.2	1.1e-01	3.7e-01
Positive Probability	0.04	0.11	0.19	0.1	1.5e-01	3.7e-01

Table S4: Summary of Adenoma based on Surgery Received

Variable	Surgery Mean	No Surgery Mean	Surgery SD	No Surgery SD	P-value	BH Corrected
Richness	1e+02	-4e+01	138.9	1e+02	5.6e-03	3.3e-02
Shannon	6e-01	-1e-01	0.9	4e-01	4.5e-02	1.3e-01
Evenness	6e-02	-9e-03	0.1	5e-02	1.0e-01	1.6e-01
Thetayc	6e-01	6e-01	0.2	2e-01	5.7e-01	6.9e-01
Adenoma Only						
Positive Probability	1e-01	1e-02	0.1	1e-01	6.6e-02	1.3e-01
Advanced Adenoma Only						
Positive Probablity	3e-02	5e-02	0.1	1e-01	8.0e-01	8.0e-01

Table S5: Summary of mtry ranges used for the treatment and diagnosis models

mtry	Occurence	Model	Disease
2	91	random	adenoma
42	9	random	adenoma
2	75	random	advanced adenoma
52	23	random	advanced adenoma
1353	2	random	advanced adenoma
2	76	random	carcinoma
41	18	random	carcinoma
863	6	random	carcinoma
2	42	treatment	adenoma
42	26	treatment	adenoma
914	32	treatment	adenoma
2	26	treatment	advanced adenoma
52	14	treatment	advanced adenoma
1353	60	treatment	advanced adenoma
2	36	treatment	carcinoma
41	54	treatment	carcinoma
863	10	treatment	carcinoma
2	16	diagnosis	adenoma
37	44	diagnosis	adenoma
707	40	diagnosis	adenoma
2	33	diagnosis	advanced adenoma
37	50	diagnosis	advanced adenoma
696	17	diagnosis	advanced adenoma
36	96	diagnosis	carcinoma
649	4	diagnosis	carcinoma
