# Supplementary figures and images for: Normalization of the microbiota in patients after treatment for colonic lesions
Source: Microbiome. 2017 Nov 16;5:150. doi: 10.1186/s40168-017-0366-3 (PMC5689185; doi:10.1186/s40168-017-0366-3)

**A**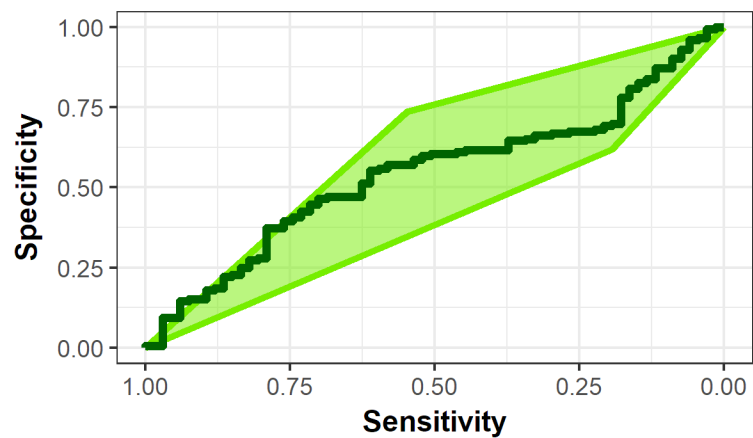**B**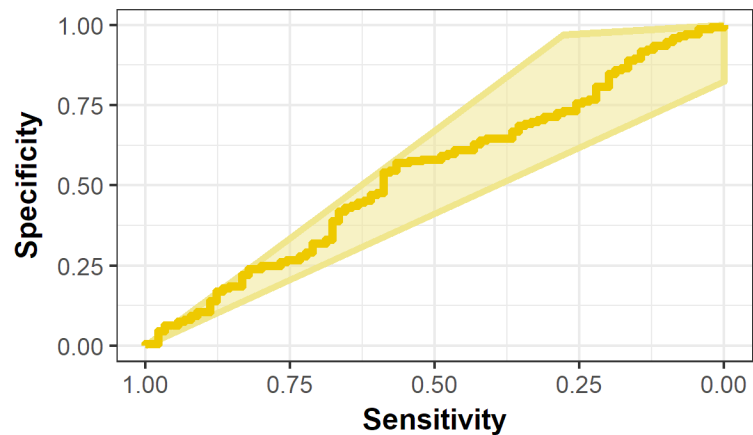**C**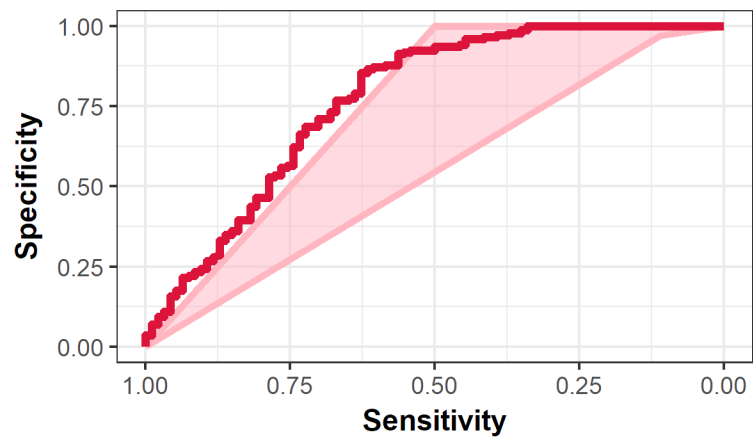

Supplement: Supplementary file 2 — Figure S1: ROC curves of the adenoma, advanced adenoma, and carcinoma models. A) Adenoma ROC curve: the light green shaded areas represent the range of values of a 100 different 80/20 splits of the test set data and the dark green line represents the model using 100% of the data set and what was used for subsequent classification. B) Advanced Adenoma ROC curve: the light yellow shaded areas represent the range of values of a 100 different 80/20 splits of the test set data and the dark yellow line represents the model using 100% of the data set and what was used for subsequent classification. C) Carcinoma ROC curve: the light red shaded areas represent the range of values of a 100 different 80/20 splits of the test set data and the dark red line represents the model using 100% of the data set and what was used for subsequent classification. (PDF 92 kb) [file 40168_2017_366_MOESM2_ESM.pdf]

**A**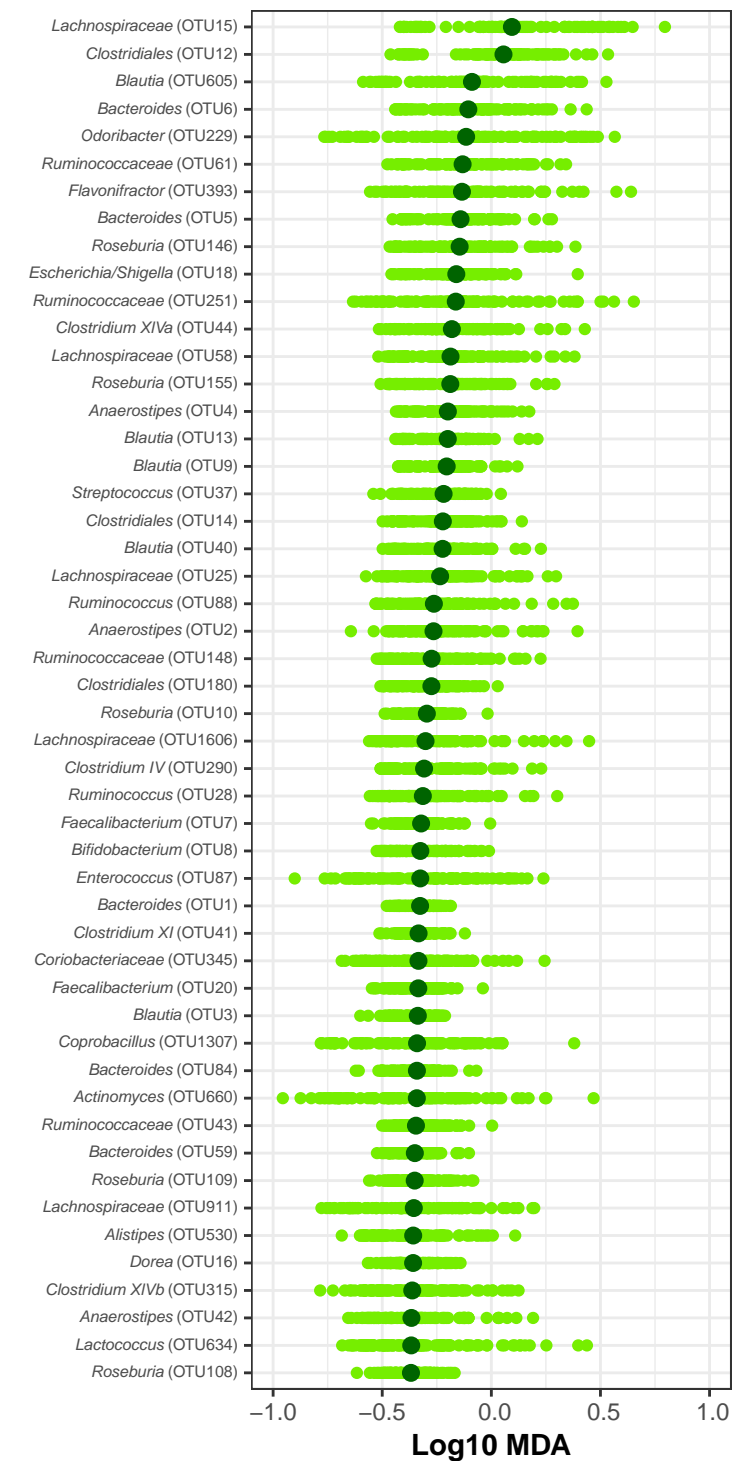**B**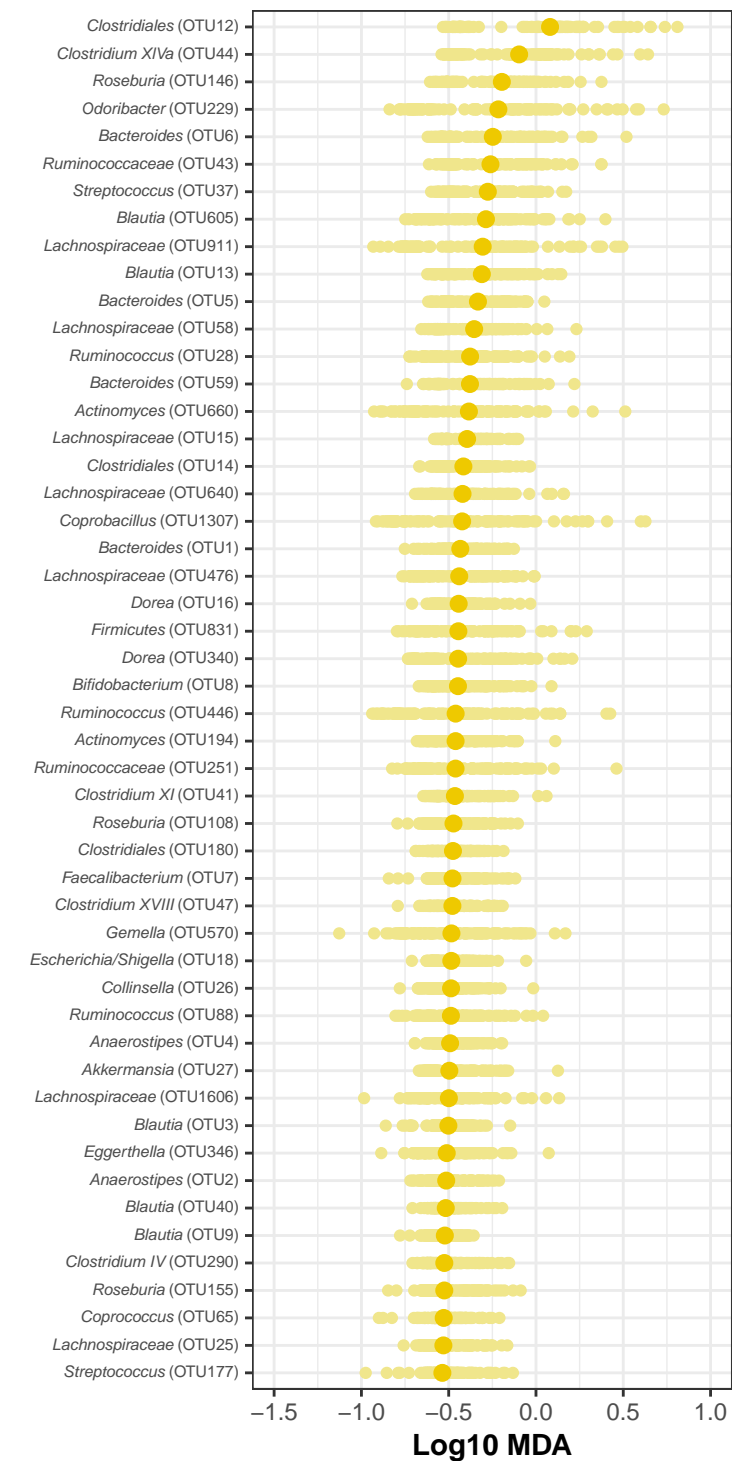**C**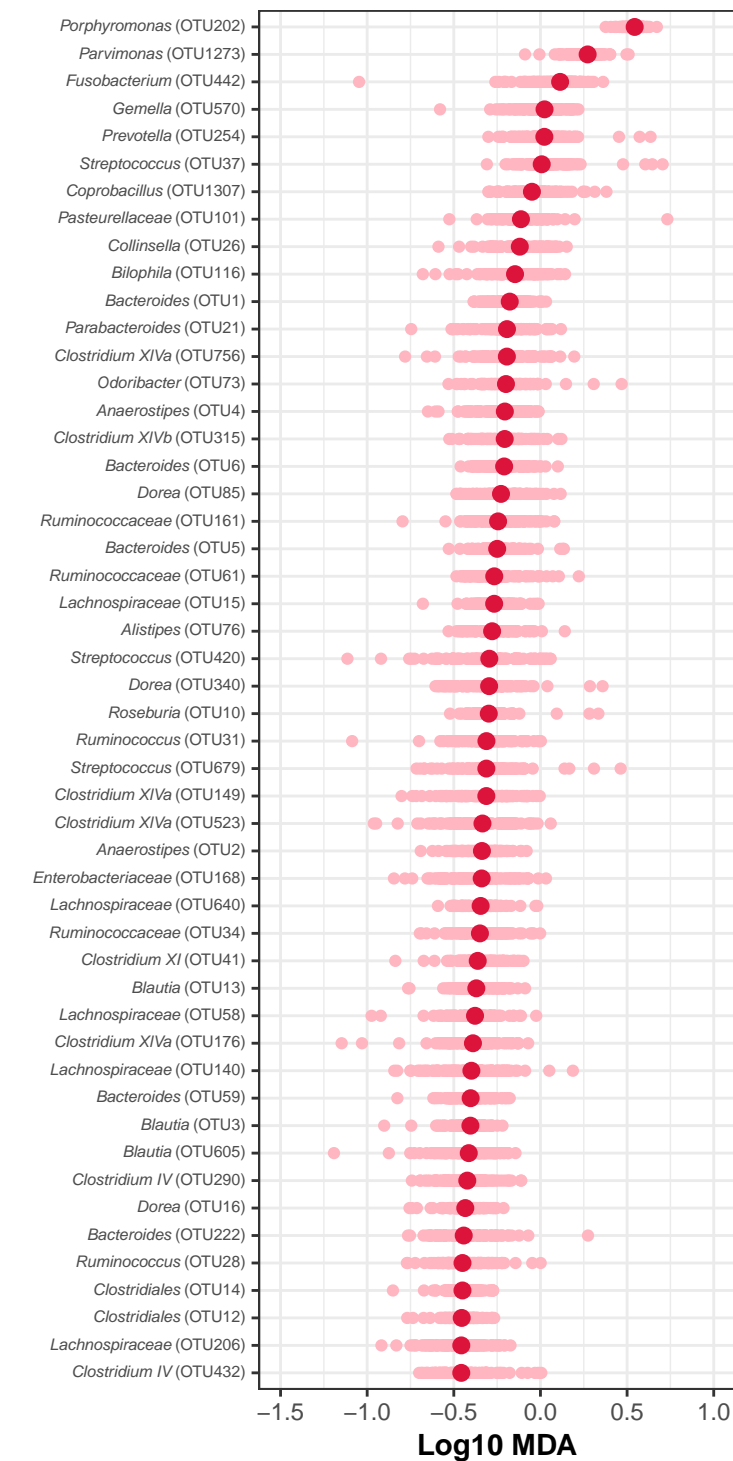

Supplement: Supplementary file 3 — Figure S2: Summary of top 10% of important OTUs for the adenoma, advanced adenoma, and carcinoma models. A) MDA of the most important variables in the adenoma model. The dark green point represents the mean and the lighter green points are the value of each of the 100 different runs. B) Summary of important variables in the advanced adenoma model. MDA of the most important variables in the SRN model. The dark yellow point represents the mean and the lighter yellow points are the value of each of the 100 different runs. C) MDA of the most important variables in the carcinoma model. The dark red point represents the mean and the lighter red points are the value of each of the 100 different runs. (PDF 87 kb) [file 40168_2017_366_MOESM3_ESM.pdf]

Relative Abundance

*Porphyromonas* (OTU202)

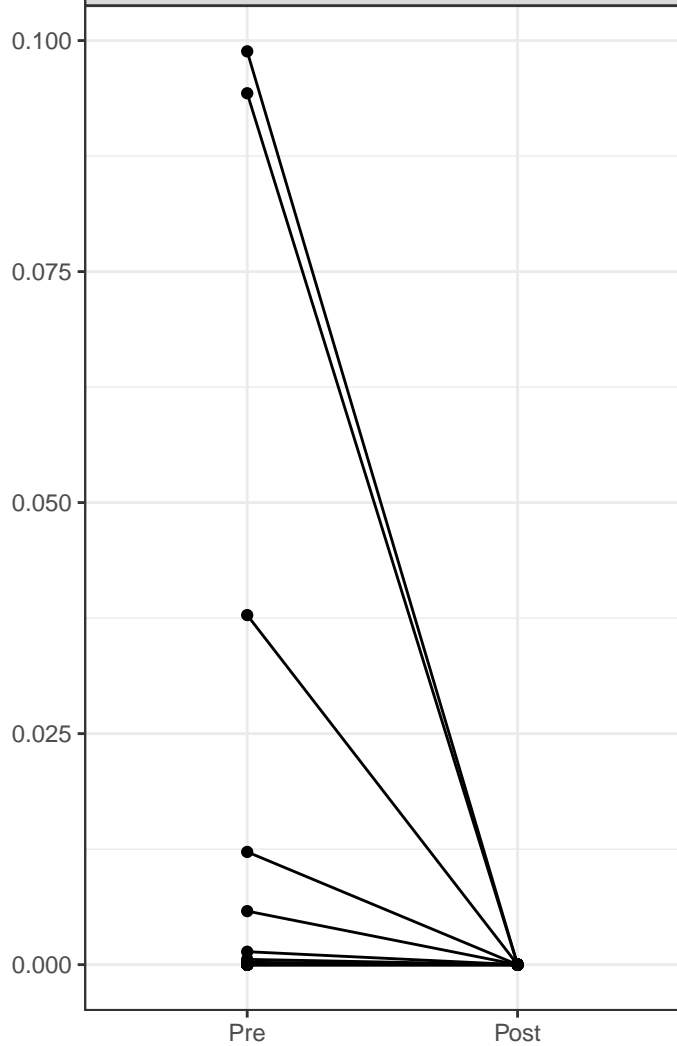

*Parvimonas* (OTU1273)

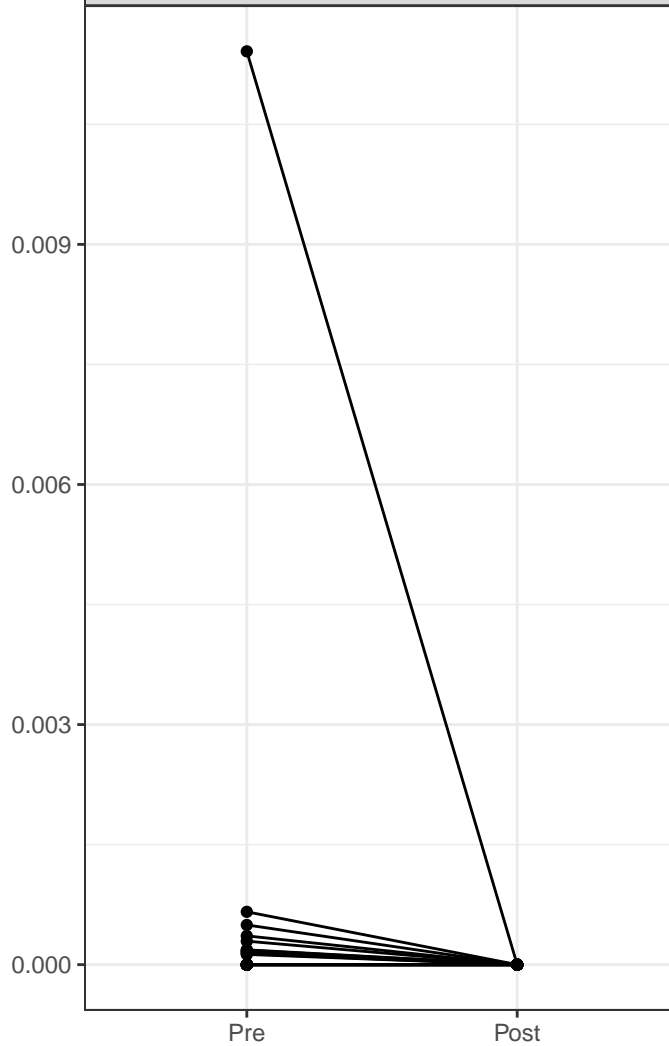

*Fusobacterium* (OTU442)

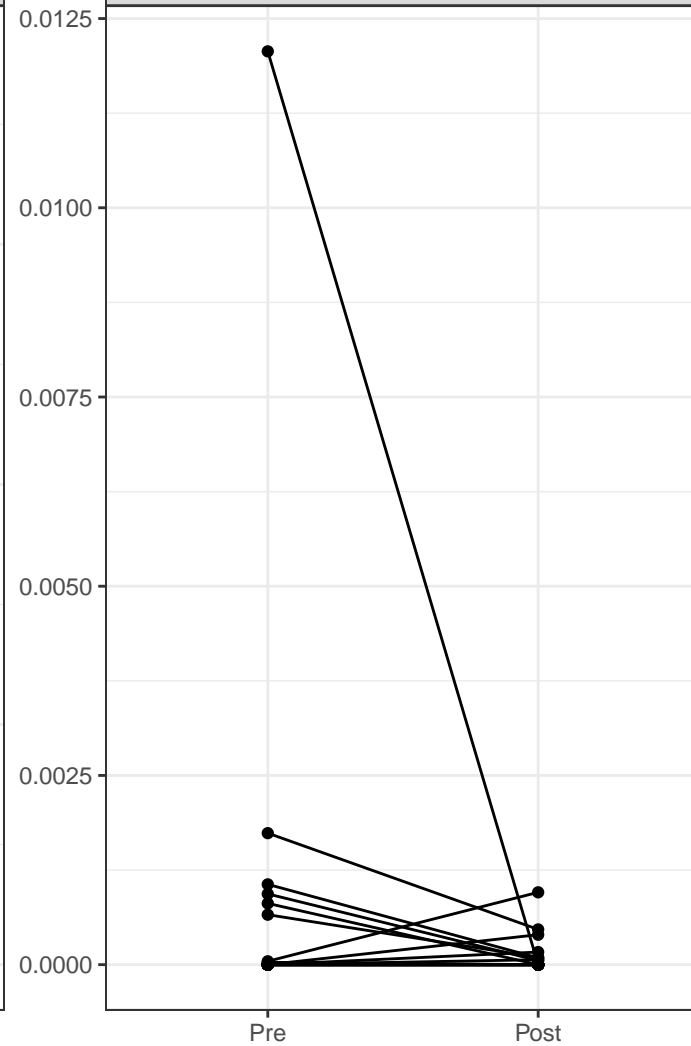

Supplement: Supplementary file 4 — Figure S3: Pre and post-treatment relative abundance of CRC associated OTUs within the carcinoma model. (PDF 6 kb) [file 40168_2017_366_MOESM4_ESM.pdf]
